# Deep language space neural network for classifying mild cognitive impairment and Alzheimer-type dementia

**DOI:** 10.1371/journal.pone.0205636

**Published:** 2018-11-07

**Authors:** Sylvester Olubolu Orimaye, Jojo Sze-Meng Wong, Chee Piau Wong

**Affiliations:** 1 Department of Biostatistics and Epidemiology, College of Public Health, East Tennessee State University, Johnson City, TN, United States of America; 2 Clayton School of Information Technology, Monash University, Melbourne, Victoria, Australia; 3 Perdana University - Royal College of Surgeons in Ireland School of Medicine, Kuala Lumpur, Malaysia; Xuanwu Hospital, Capital Medical Universty, CHINA

## Abstract

It has been quite a challenge to diagnose Mild Cognitive Impairment due to Alzheimer’s disease (MCI) and Alzheimer-type dementia (AD-type dementia) using the currently available clinical diagnostic criteria and neuropsychological examinations. As such we propose an automated diagnostic technique using a variant of deep neural networks language models (DNNLM) on the verbal utterances of affected individuals. Motivated by the success of DNNLM on natural language tasks, we propose a combination of deep neural network and deep language models (D2NNLM) for classifying the disease. Results on the DementiaBank language transcript clinical dataset show that D2NNLM sufficiently learned several linguistic biomarkers in the form of higher order *n*-grams to distinguish the affected group from the healthy group with reasonable accuracy on very sparse clinical datasets.

## Introduction

Early diagnosis of Mild Cognitive Impairment due to Alzheimer’s disease (MCI) and Alzheimer-type dementia (AD-type dementia) is currently a challenge [1] [2]. More importantly, both MCI and AD-type dementia have been typically diagnosed through extensive neuropsychological examinations using a series of cognitive tests containing a set of questions and images [3]. For example, the Mini-Mental State Examination (MMSE) and the Montreal Cognitive Assessment (MoCA) screening tools are composed of a series of questions and cognitive tests, that assess different cognitive abilities. The challenges with these cognitive tests are that the accuracy depends on the clinician’s level of experience and their ability to diagnose different sub-types of the disease [4]. Often, researchers and clinicians need to combine other cognitive tests with the MMSE [5], and in most cases wait for a reasonably long interval to ascertain the diagnosis [6]. More recently, research has also shown that the reliability of the MMSE as a tool for diagnosing AD-type dementia could be limited [7]. The National Institute on Aging and the Alzheimer’s Association has also called for several other clinical criteria that could be used to effectively diagnose MCI and AD-type dementia and other similar disease in a non-invasive way [8].

As opposed to the ad hoc use of neuropsychological examinations, linguistic ability captured from verbal utterances could be a good indication of Mild Cognitive Impairments due to AD-type dementia (MCI) and AD-type dementia [9]. The premise is that both MCI and AD-type dementia are characterized by the gradual deterioration of nerve cells that control cognitive, speech and language processes, which consequentially translates to how patients compose verbal utterances [10–12]. According to [13], syntactic processing in acquired language disorders such as Aphasia in adults has shown promising findings, encouraging further study of identifying effective syntactic techniques. Similarly, [14] emphasized the significance of lexical-semantic components of a language, part of which is observable during the utterance acquisition at a younger age. That work further highlighted that as the lexical capacity increases, syntactic processing becomes automated, which leads to lexical and syntactic changes in language.

As such, we are motivated by the effectiveness of deep neural networks language models (DNNLM) in modeling acoustic signals for clinical natural language tasks [15]. We explore deep-deep neural networks language models (D2NNLM) to learn the linguistic changes that distinguish the language of patients with MCI and AD-type dementia from the healthy controls using higher order *n*-grams. The ordinary DNNLM uses lower order *n*-gram *N*-dimensional sparse vectors as discrete feature representations to train the neural network with multiple hidden layers [15]. In this paper, we maintain the same deep neural network (DNN) architecture and increase the depth of the language models by introducing higher order *n*-gram *N*-dimensional sparse vectors as discrete inputs to the DNN rather than single word *N*-dimensional sparse vectors. In other words, we create *n*-gram vocabulary spaces from which we form the *N*-dimensional sparse vectors. The premise is that clinical datasets are usually sparse and it is the same for the DementiaBank (https://dementia.talkbank.org) dataset used in this paper [16]. Thus, using lower order *n*-gram dimensional sparse vectors alone could limit the vocabulary space and subsume the essential linguistic changes and biomarkers, which could potentially distinguish patients with MCI and AD-type dementia from the healthy controls. On the other hand, higher order *n*-grams have been shown to be good class predictors in several language modeling tasks on sparse data [17]. To the best of our knowledge, little work has considered deep neural network and deep language models for classifying MCI and AD-type dementia on sparse clinical language datasets.

## Related work

In [18], the efficacy of using complex syntactic features to classify MCI was demonstrated. In that work, spoken language characteristics were used to discriminate between 37 patients with MCI and 37 in the healthy elderly group using 7 significant pause and syntactic linguistic annotations as features to train Support Vector Machines (SVM). That technique achieved 86.1% Area Under the ROC Curve (AUC). In contrast, we use language models, which are more representative of the language space of both the disease and healthy groups without using any handcrafted features.

More recently, [19] proposed a ‘graph-based content word summary score’ and a ‘graph-based content word word-level score’ to classify AD-type dementia, which is often preceded by MCI [18]. Using SVM on the same DementiaBank dataset, that work achieved 82.3% AUC. However, the graph-based techniques require separately built alignment models with sufficiently large datasets.

This paper has two main contributions. (1) We introduce deep language models in the form of decomposed higher order *n*-grams *N* dimensional vectors as discrete inputs to the DNN, hence we derived D2NNLM. (2) We show that D2NNLM predicts MCI and AD-type dementia with less percentage error, perplexity, and AUC, especially on sparse clinical language datasets.

## Deep neural network language models

The DNNLM architecture has more than one hidden layer with nonlinear activations [15], and it is built on top of the original feed-forward neural network language model (NNLM) architecture [20]. Unlike the DNNLM, NNLM has only two hidden layers. The first hidden layer has a linear activation and often referred to as the projection layer. The second hidden layer uses a non-linear activation, hence making the NNLM a single hidden layer neural network [20].

In this paper, we follow the notations used in [15] and [21] to describe the components of the DNNLM architecture. Given a vocabulary space, each word in the vocabulary is denoted by an *N*-dimensional sparse vector. In each vector, the index of that particular word is stored with 1 while other indices in the vector are stored with 0s. As inputs to the neural network, the discrete feature representations are concatenated to contain the *n*-1 previous words in the vocabulary space, which serves as the memory to the previous words history. Given that *N* is the vocabulary size and *P* is the dimension of the continuous feature space, linear projections of all the concatenated words are used to create the first hidden layer of the network from every *i*th row of the *N* x *P* dimensional projection matrix. This is followed by the hidden layer *H* with hyperbolic tangent non-linear activation functions as follows:

$$\begin{array}{c} d_{j}=\text{tanh}(\sum_{l=1}^{(n-1)\times P}M_{jl}c_{l}+b_{j})\forall j=1,\ldots ,H \end{array}$$ (1)

where the weights between the projection layer and the subsequent hidden layers are denoted with *M*_*jl*_, and the biases of the hidden layers are represented with *b*_*j*_. Note that the hyperbolic tangent non-linear activation function has been shown to converge quickly and effectively in Language Models [20].

Note that since the DNNLM follows the NNLM architecture, other hidden layers with the same hyperbolic tangent non-linear activation functions are added to make the network deeper. The output layer uses a softmax function to simultaneously compute the language model probability of each word *i* giving its history, *h*_*j*_, thus *P*(*w*_*j*_ = *i*|*h*_*j*_). We present the details of the output layer and the language model probability as follows:

$$\begin{array}{c} o_{i}=\sum_{j=1}^{H}V_{ij}d_{j}+k_{i}\forall i=1,\ldots ,N \end{array}$$ (2)

$$\begin{array}{c} p_{i}=P\left(w_{j}=i|h_{j}\right)=\frac{\text{exp}(o_{i})}{\sum_{l=1}^{N}\text{exp}\left(o_{l}\right)}\forall i=1,\ldots ,N \end{array}$$ (3)

where *V*_*ij*_ denotes the weights between the hidden layers and the output layer, *k*_*i*_ represents the biases of the output layer and the *p*_*i*_ computes the language model probability for every *i*th output neuron.

## Deep-deep neural network language models

**Fig 1:**
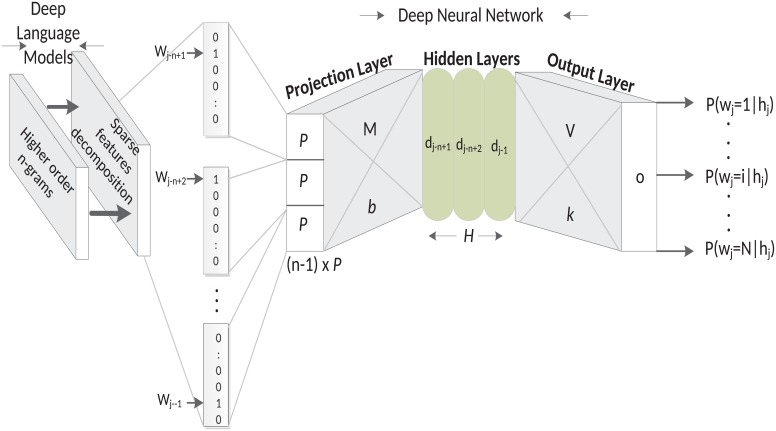
Deep-deep neural network language models.

Though the D2NNLM uses a somewhat different architecture compared to the DNNLM, nevertheless, the D2NNLM is comprised of multiple hyperbolic tangent non-linear activation functions. On top of that, we make the vocabulary space deeper by increasing the *n*-gram vocabulary space that was used in the original DNNLM and also use a decomposition technique to reduce the sparse feature space to a lower dimensional feature space in order to minimize the generalization error [22]. Fig 1 shows the architecture of the D2NNLM.

With regard to classifying language utterances with symptoms of MCI and AD-type dementia, it is of paramount importance to our D2NNLM that the language is modeled with a vocabulary space of substantial depth due to the non-trivial nature of the problem [18, 23]. For example, according to the study conducted by [18], many of the handcrafted language and speech measures that have been used in distinguishing patients with MCI from their respective healthy controls—including some statistically significant measures—have shown the means and the standard deviations to be very close between the MCI and the healthy control groups. Thus, it is probable that very little linguistic deficits will characterize either group. Even with DNNLM, which is based on simple *n*-gram language models with embedded words as a continuous feature space, it is still challenging to generalize over unseen data due to the data sparseness problem [15]. As such, an alternate technique could be found in using deeper language models with higher order *n*-grams for embedding the vocabulary in a much deeper continuous language space [24]. As shown in Fig 1, we used higher order *n*-gram language models to create deep language models for deep neural network. We refer to such models as Deep-Deep Neural Network Language Models and our preliminary experiments show that deeper language models potentially improve the performance of the deep neural network for classifying MCI and AD-type dementia. We will describe the generation of the *n*-gram vocabulary space in the following section.

Similar to DNNLM, the computational complexity of D2NNLM could be characterized by the undecomposed feature space, which typically has a large but sparse matrix containing a few 1s and a lot of 0s. As such, rather than using hierarchical decomposition of output probabilities of the neural network [15], we performed Singular Value Decomposition (SVD) [22], to produce a reduced-rank approximation of the higher order *n*-gram vocabulary spaces before mapping the vocabularies into the continuous feature space, which represents the projected vocabulary history vectors. We believe that SVD becomes a straightforward option to produce a compact approximation of the original feature space with optimal least-square as it sufficiently models the frequently occurring 0s, which are often not informative [24]. In other words, the distinguishing deep language space features are still preserved, while noisy features are concealed. Thus, only the low-dimensional vocabulary is used to learn the output targets. Finally, we followed [25] and trained the neural network using the standard back-propagation algorithm to minimize the error function and reduce overfitting. According to [25], one can train Deep Neural Networks discriminatively using the backpropagating derivatives of the error function which measures the difference between the target probabilities and the actual probabilities. The error function *E*_*r*_ is computed as follows:

$$\begin{array}{c} E_{r}=\sum_{i=1}^{N}t_{i}\;\text{log}\;p_{i}+\epsilon (\sum_{jl}M_{jl}^{2}+\sum_{ij}V_{ij}^{2})\forall j=1,\ldots ,H \end{array}$$ (4)

where *t*_*i*_ is the target vector, parameter *ϵ* is determined empirically using the validation set. Note that the first half of the equation computes the cross entropy between the output and the target probability masses and the second half computes the regularization term, which avoids overfitting the training data [25].

### *n*-gram vocabulary space

The use of word *n*-gram is popular in Natural Language Processing (NLP) tasks, especially for developing language models that are able to characterize the lexical usage of grammar in a dataset [26]. A word *n*-gram is the sequence of words identified as an independent representation of a part of the grammar in an utterance or a sentence. ‘*n*’ in this case represents the number of words in the sequence. For instance, when *n* is 1, it is called a ‘unigram’, which has only one word. Similarly, a ‘bigram’ and a ‘trigram’ have *n* equal to 2 and 3 respectively, and it is not uncommon to use higher order *n*-grams (i.e. *n* ≥ 3) in learning tasks [24]. In this paper, our *n*-gram vocabulary space consist of 4 and 5 *n*-grams, which are generated from the transcripts of both the disease and the healthy control groups. Moreover, the 4-gram and 5-gram features subsume other lower order *n*-grams such as unigrams, bigrams, and trigrams [24]. We put emphasis on higher order *n*-grams because they are known to have performed with reasonable accuracy in other NLP and ML tasks [27].

## Experiment and results

### Dataset

We performed experiments on the clinical Pitt Corpus of the DementiaBank (https://dementia.talkbank.org/access/English/Pitt.html) [16]. The dataset was created during a longitudinal study conducted by the University of Pittsburgh School of Medicine on Alzheimer’s disease and related Dementia and was funded by the National Institute of Aging (http://www.nia.nih.gov/). The dataset contains transcripts of verbal interviews with healthy controls and patients that were diagnosed with MCI and variants of AD-type dementia (e.g. probable and definite AD) using the Mini-Mental State Examination (MMSE) and other clinical diagnostic measures [16].

According to [27], all the participants in the DementiaBank dataset enrolled in the Alzheimer Research Program between March 1983 and March 1988. The final classification of the participants was made based on a consensus clinical diagnosis, neuropathologic diagnosis, and an autopsy at the death after about 5 years of follow-up. After follow-up, the accuracy of the clinical diagnosis of the AD-type dementia was 91.4% with a high sensitivity of 98.8% and specificity of 98.3%.

Interviews were conducted in the English language and were based on the description of the Cookie-Theft picture component, which is part of the Boston Diagnostic Aphasia Examination. During the interview, patients were given the picture and were told to discuss everything they could see happening in the picture. The patients’ verbal utterances were recorded and then transcribed into a transcription format with the equivalent text.

With the complete demographic details presented in [16], we selected 99 transcripts of patients with the probable AD as the AD-type dementia group. That group has an age range of 55 to 90 years. We selected all the available 99 controls which are healthy elderly individuals without any reported diagnosis. The healthy control group has an age range of 46 to 81 years. The MCI group has 19 patients between ages 49 and 81 years with a matching control of 19 healthy individuals who were randomly selected from the available 99 control individuals. The 19 healthy control individuals have an age range of 55 to 74 years.

### Baselines

We compared our work with three different baselines. First, in order to show the efficacy of our deep language model and deep neural network technique, we compared our model to the DNNLM [15], and the original NNLM [20], as the neural network baselines on the DementiaBank dataset. As discussed earlier, the D2NNLM and the two neural network baselines have different architectures but used the same learning and regularization settings. At the same time, each model used the optimal number of hidden units as identified by the grid search technique and the hidden layers in each model used the same number of optimal hidden units. The D2NNLM (3 hidden-layers) takes the left-singular matrix of the SVD from 4-grams and 5-grams as inputs respectively, while the DNNLM (3 hidden layers) and the NNLM (1 hidden layer) take as input only the original sparse feature representation of 4-grams as performed in [15]. Note that the DNNLM and the NNLM are considered to have performed more than the conventional *n*-gram language model such as 4-gram [27], which does not have a neural network architecture.

The other baselines include the conventional 4-gram language model, [18], which used different handcrafted speech measures from self-collected clinical datasets, and [19], which used word alignment features.

In [18], the Wechsler Logical Memory task [28], was used to collect language and speech data from a narrative memory task, which required 32 patients with MCI and 32 control individuals to listen to a story and then recall everything they can from the story. That task allowed the subjects to formulate original language structures on their own. It also helps to capture both linguistic and memory deficiencies from the subjects by using various language and speech measures. As such, we implemented all the 7 Wechsler Logical Memory I significant features from [18] and test on our dataset. Those features include Words per clause, Part-Of-Speech cross entropy, content density, Standard Pause Rate, Total Phonation Time, Phonation Rate, and Transformed Phonation Rate. Although the language and speech data from the DementiaBank dataset was collected differently from the Wechsler Logical Memory task in [18]; nevertheless, the Cookie-Theft picture description task in the DementiaBank dataset required the subjects to also formulate their language structures by describing the scenes on the picture in no particular order. This is quite important in diagnosing patients with MCI and AD-type dementia as many linguistic defects will likely show in the inability of the patients to describe the scenes in a meaningful order. Also, we did not consider the Wechsler Logical Memory II significant features in [18] as a baseline because that task captures much longer memory deficiencies by making the subjects recall the story after 30 minutes or more. Our goal was to detect immediate linguistic deficiencies from the subjects, which could aid quick diagnostics rather than delayed.

### D2NN language models settings

We generated the vocabulary for the D2NNLM from each dataset. The MCI dataset contains 210 sentences from patients with MCI and 236 sentences from healthy controls. On the other hand, the AD-type dementia dataset has a total of 1392 sentences and a total of 1236 sentences from the 99 control individuals. Table 1 shows the details of the *n*-gram vocabularies, which were generated from the two datasets. As shown in Table 2, the D2NNLM training data consist of 50% of each dataset’s transcript files, while each of the test and validation sets consist of 25% of the transcript files.

**Table 1:** Details of *n*-gram vocabularies from the MCI and AD-type dementia datasets.

Vocabulary	4-grams	5-grams
MCI	841	748
Control	915	806
Total *n*-grams	1756	1554
Unique *n*-grams	1642	1508
AD-type dementia	3781	3264
Control	4054	3533
Total *n*-grams	7835	6797
Unique *n*-grams	6948	6475

**Table 2:** Percentages of transcript files for training, test, and validation sets for the MCI and AD-type dementia datasets.

Dataset	MCI/Control	AD-type dementia/Control
Training	50%	50%
Test	25%	25%
Validation	25%	25%
Total (size)	100% (38)	100% (198)

The decomposed left-singular matrix from the SVD maps the *n*-gram vocabulary histories into a lower dimensional continuous parameter space for the D2NNLM. This generated 19 lower dimensional features per instance from the MCI dataset and 198 lower dimensional features per instance from the AD-type dementia dataset. Using the Theano Python library(http://deeplearning.net/software/theano/), we implemented the D2NNLM and our baselines as Multilayer Perceptron (MLP) with respective hidden layers as appropriate. We trained the D2NNLM with three hidden layers and a projection layer. The D2NNLM performs a classification task by discriminating between the respective disease and the control classes using stochastic gradient descent optimization with different minibatches [25]. We investigated 10 different batch sizes for the MCI dataset and 50 different batch sizes for the AD-type dementia dataset since it has more feature dimension and number of instances.

For the classification task, the network parameters are used to estimate the likelihood that a vocabulary feature sequence belongs to either the MCI or Control class for the MCI dataset, and either the AD-type dementia or Control class for the AD-type dementia dataset. Unlike [15], we set the number of epochs to 500 and used the regularization implementation in Theano to enforce the L1 and L2 regularization parameters to small values that are close to zero as recommended in [29].

Finally, we performed a grid search to identify the optimal number of hidden units from the minibatch with the lowest percentage Mean Square Error (MSE) on the held-out test set and validation set. The percentage error (or MSE) is used often to evaluate neural network models [15]. Using the optimal number of hidden units for the hidden layers avoids the risk of reconstructing the identity function for the neural network [30]. We also estimated the language model perplexity of the D2NNLM in comparison to DNNLM and NNLM. In language modeling, perplexity measures how well a model predicts given examples using an information theoretic approach [20]. A better model minimizes the perplexity. We compute the perplexity as 2^*B*(*q*)^ as follows:

$$\begin{array}{c} B\left(q\right)=-\frac{1}{N}\sum_{i=1}^{N}log_{2}q\left(x_{i}\right) \end{array}$$ (5)

$$\begin{array}{c} Perplexity=2^{B(q)} \end{array}$$ (6)

where *B*(*q*) estimates the cross-entropy or the negative log-likelihood of the model which shows the ability of the model to predict a significant portion of the test samples.

### Results

First, we analyze our results in comparison to our neural network baselines. As shown in Tables 3 and 4, we performed experiments by comparing the percentage error and perplexity between D2NNLM, DNNLM, and NNLM. The D2NNLM has two variants, one with decomposed 4-gram features (D2NNLM-4n) and the other with decomposed 5-gram features (D2NNLM-5n).

**Table 3:** % error and perplexity on MCI held-out test set. (h = Hidden layer size; Bz = Batch size).

Models	(%) Error	Perplexity
D2NNLM-4n (n = 4,h = 11,Bz = 9)	**11.1**	**1.5**
D2NNLM-5n (n = 5,h = 19,Bz = 6)	**16.7**	**1.4**
DNNLM (n = 4,h = 300,Bz = 5)	20.0	1.6
NNLM (n = 4,h = 150,Bz = 4)	25.0	1.6

**Table 4:** % error and perplexity on AD-type dementia held-out test set (h = Hidden layer size; Bz = Batch size).

Models	(%) Error	Perplexity
D2NNLM-4n (n = 4,h = 7,Bz = 29)	**24.1**	**1.5**
D2NNLM-5n (n = 5,h = 127,Bz = 35)	**25.7**	**1.5**
DNNLM (n = 4,h = 300,Bz = 34)	26.5	1.6
NNLM (n = 4,h = 300,Bz = 20)	27.5	1.5

On the MCI dataset, the D2NNLM-4n achieved a low percentage error of 11.1% and reduced perplexity of 1.5 with 11 hidden units per hidden layer and training with 9 batch size. Similarly, D2NNLM-5n used 19 hidden units and has a reduced error of 16.7% with perplexity at 1.4. On the other hand, the DNNLM achieved its lowest percentage error with 300 hidden units and 5 batch size. Similarly, NNLM used 150 hidden units and 4 batch size for its lowest percentage error.

On the AD-type dementia dataset, the D2NNLM-4n showed a better percentage error of 24.1% and a perplexity of 1.5 compared to the D2NNLM-5n with 25.7% error and the same 1.5 perplexity, DNNLM with 26.5% error and 1.6 perplexity, and NNLM with 27.5% error and 1.5 perplexity.

**Fig 2:**
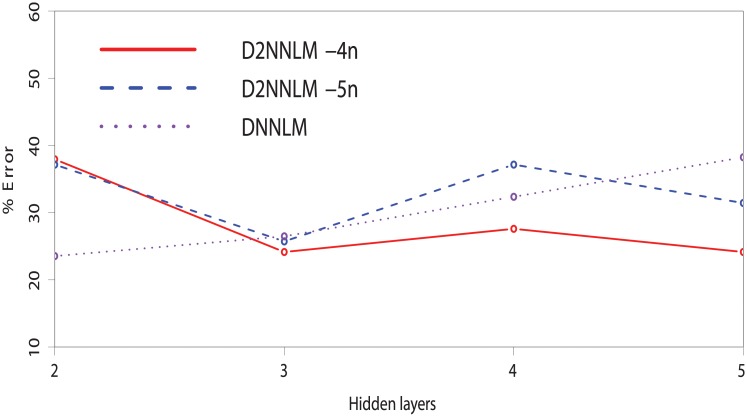
% Error of the D2NNLMs vs. DNNLM on MCI dataset with smaller number of hidden layers.

**Fig 3:**
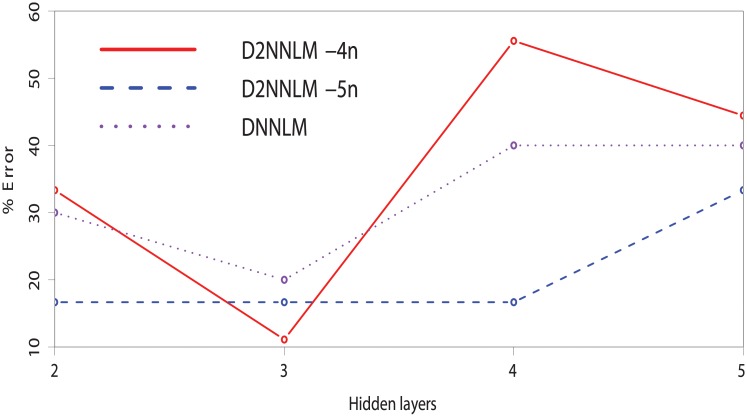
% Error of the D2NNLMs vs. DNNLM on AD-type dementia dataset with smaller number of hidden layers.

As shown in Figs 2 and 3, we investigated the effect or robustness of smaller number of hidden layers on the D2NNLM-4n, D2NNLM-5n, and DNNLM by varying the hidden layers from 2 to 5 on both datasets. Note that we did not include the single-layer because it will be the same as NNLM, thus subsequent layers create a deeper architecture for the models. Interestingly, D2NNLM-4n gave a better performance with the lowest percentage error plot at the third hidden layer on the MCI dataset. However, the performance of D2NNLM-4n degrades significantly at the fourth and fifth hidden layers. Nevertheless, the D2NNLM-5n showed a more robust and consistent performance on the MCI dataset with lower number of hidden layers, giving the same percentage error of 16.7% across hidden layers 2, 3, and 4, respectively. Even at the fifth hidden layer, the D2NNLM-5n only moved slightly above 30%.

**Fig 4:**
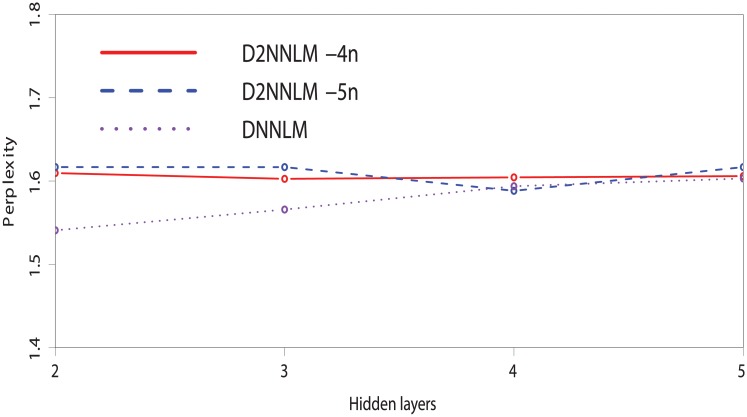
Perplexity comparison between the D2NNLMs and DNNLM on the MCI dataset with smaller number of hidden layers.

**Fig 5:**
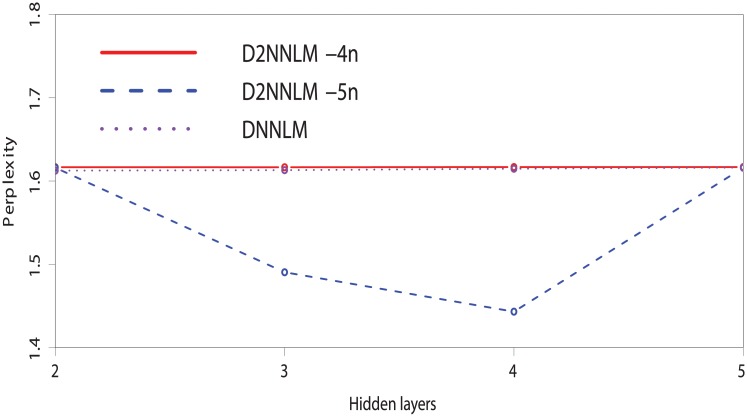
Perplexity comparison between the D2NNLMs and DNNLM on the AD-type dementia dataset with smaller number of hidden layers.

Similarly, Figs 4 and 5 compare the perplexities between the D2NNLM-4n, D2NNLM-5n, and DNNLM with smaller number of hidden layers. We observed that the D2NNLM-5n showed a plot with lower perplexity on the MCI dataset than the D2NNLM-4n at the third and fourth hidden layers with 1.45 and 1.50 perplexities. Similarly on the AD-type dementia dataset, the D2NNLM-5n showed a lower perplexity of 1.58 at the fourth hidden layer than the D2NNLM-4n, which maintains perplexities above 1.60 across all the hidden layers. DNNLM showed lower perplexities on the AD-type dementia dataset at hidden layers 2 and 3 but has higher error rate than the D2NNLM-4n and D2NNLM-5n.

### Discussion

We see that the D2NNLM-4n and D2NNLM-5n have better percentage errors and mostly lower perplexities than those achieved by DNNLM and NNLM on both datasets. On the other hand, we observed that D2NNLM-5n has a lower performance than D2NNLM-4n. Although, the performance of D2NNLM-5n is still better than DNNLM and NNLM, we observed that an optimal model with better generalization error could be achieved by using D2NNLM-4n, while D2NNLM-5n gives a good language model with lesser perplexity.

In terms of the robustness of our models with smaller number of hidden layers, we observed a slight difference in performance on the AD-type dementia dataset as the D2NNLM-4n gave a lower percentage error at both the third and fifth hidden layers. Unlike the MCI dataset, the D2NNLM-5n showed its lowest percentage error at hidden layer 3, while the D2NNLM-4n gave a more robust and consistent performance across hidden layers 3, 4, and 5. It is likely that this evidence indicates the difference between the MCI and AD-type dementia research problems [11, 18, 19]. Much longer word dependencies may be needed to capture the linguistic deficits from patients with MCI because they mostly exhibit similar linguistic characteristics as the healthy control individuals [18]. On the other hand, patients with AD-type dementia are likely to show pronounced linguistic deficits, which can be easily captured by the D2NNLM-4n without stretching the language model. Although the resulting percentage errors on the AD-type dementia are higher than those of the MCI, both D2NNLM-4n and D2NNLM-5n gave mostly better percentage errors than the DNNLM across hidden layers 3, 4, and 5.

As such, we believe that these results show the importance of higher order *n*-gram towards the deep-deep neural network language model for classifying MCI and AD-type dementia. We also believe that the increased feature dimension might have led to the improved performance [20], which emphasizes the importance of a much deeper language model vocabulary space with higher order *n*-gram features.

We evaluated the clinical relevance of our models with the AUC similar to [18] and [19]. Using the same grid search technique, we compared the performance of the D2NNLM-4n and D2NNLM-5n with the 7 significant speech measures in [18] and a 4-gram conventional language model [27]. We estimated the AUC using the same leave-pair-out cross-validation (LPOCV) evaluation that was used in [18]. Note that, the LPOCV produces an unbiased estimate of the AUC for clinical diagnostics [18, 19]. Also, choosing LPOCV as a reliable clinical evaluation technique has been extensively argued in the literature [31, 32]. Unlike other evaluations, every pair of positive and negative example is evaluated on a model trained on the rest of the examples. For example, from the 99 AD-type dementia patients and 99 control individuals, each round of the LPOCV selects a unique pair of one patient with AD-type dementia and one control individual as the test set for evaluating a model trained with the remaining 98 patients with AD-type dementia and 98 control individuals. The same evaluation is also applicable to the MCI datasets. The evaluation score is the classifier’s confidence *c*, computed for each example in the test example pair, and is used to compute the Wilcoxon-Mann-Whitney statistic as follows:

$$\begin{array}{c} c\left(p,n\right)=\{\begin{array}{cc} 1 & \text{if}\;c(p)>c(n)\\ 0 & \text{otherwise} \end{array} \end{array}$$ (7)

$$\begin{array}{c} AUC\left(c,P,N\right)=\frac{1}{\left|P||N\right|}\sum_{p\in P}\sum_{n\in N}c\left(p,n\right) \end{array}$$ (8)

where *c*(*e*) is the classifier’s prediction score for an example *e*, *P* is a set of positive examples (AD-type dementia or MCI), and *N* is a set of negative examples (Control). We compute the variance of the AUC and then report the standard deviation (s.d) similar to [18] and [19]. The variance $\sigma_{AUC}^{2}$ is computed as the ratio of $A\left(1-A\right)+\left(|P|-1\right)\left(\frac{A}{2-A}-A^{2}\right)+\left(|N|-1\right)\left(\frac{2A^{2}}{1+A}-A^{2}\right)$ to |*P*||*N*|, where *A* denotes the AUC for simplicity of the expression. To obtain the statistical significance of the AUC, we calculated the p-values and the 95% confidence interval (CI)(*p* <0.05) of the AUC by converting the s.d to standard error (SE) [33].

Tables 5 and 6 show the performance comparison with the LPOCV AUC on both MCI and AD-type dementia datasets with statistical significance. We see that the D2NNLM-5n and D2NNLM-4n are more effective in classifying both groups. Compared to other techniques on the same datasets, D2NNLM-5n has a statistically significant AUC of 0.80 on the MCI dataset (*p* <0.001, CI: 0.355-1.000) and a statistically significant AUC of 0.83 on the AD-type dementia dataset (*p* <0.001, CI: 0.635-1.000). Similarly, D2NNLM-4n has a statistically significant AUC of 0.74 on the MCI dataset (*p* = 0.002, CI: 0.263-1.000) and a statistically significant AUC of 0.79 on the AD-type dementia dataset (*p* <0.001, CI: 0.581-0.999). The performance improvement over DNNLM is consistent with the percentage errors reported in Tables 3 and 4, which reaffirms the importance of a much deeper language model [20]. Also, both D2NNLM-5n and D2NNLM-4n showed improvements over the conventional 4-gram LM and the handcrafted speech measures baselines.

**Table 5:** Performance comparison with the LPOCV AUC on the MCI dataset, N = 38.

Models	AUC	s.d	SE	*p*	95% CI of AUC
D2NNLM-4n	**0.74**	**1.50**	**0.24**	**0.002**	**0.263 to 1.000**
D2NNLM-5n	**0.80**	**1.40**	**0.23**	**<0.001**	**0.355 to 1.000**
DNNLM	0.68	1.60	0.26	0.009	0.171 to 1.000
4-gram LM	0.61	1.70	0.28	0.027	0.069 to 1.000
Speech Measures	0.47	1.80	0.29	0.107	-0.102 to 1.000
Word Alignment	0.63	1.70	0.28	0.022	0.089 to 1.000

**Table 6:** Performance comparison with the LPOCV AUC on the AD-type dementia dataset, N = 198.

Models	AUC	s.d	SE	*p*	95% CI of AUC
D2NNLM-4n	**0.79**	**1.50**	**0.11**	**<0.001**	**0.581 to 0.999**
D2NNLM-5n	**0.83**	**1.40**	**0.10**	**<0.001**	**0.635 to 1.000**
DNNLM	0.73	1.60	0.11	<0.001	0.507 to 0.953
4-gram LM	0.72	3.60	0.26	0.005	0.218 to 1.000
Speech Measures	0.73	3.50	0.25	0.003	0.242 to 1.000
Word Alignment	0.68	3.70	0.26	0.010	0.165 to 1.000

In comparison to the word alignment technique proposed in [19], our models showed better AUC on our datasets, although those authors did not use the same set of MCI and AD-type dementia patients in our dataset. More importantly, unlike [19], we did not limit the participants’ descriptions to a certain number of words, rather, we used the entire description per participant. As such, our results demonstrate the potentials of D2NNLMs for classifying MCI and AD-type dementia without any handcrafted features or manually annotated datasets.

### Limitations

A limitation of this study could be the limited size of the datasets, which is often a challenge in clinical research. We believe that an increase in the data sample is likely to improve the performance of our proposed models for classifying MCI and AD-type dementia from the healthy controls.

It is worth mentioning that the use of higher order *n*-gram features in this study are limited to the description of the Cookie-Theft picture in the DementiaBank clinical dataset. This is understandable since the objects within the picture dictate the specific *n*-grams within the language space of the MCI, AD-type dementia, and control individuals. Unless a picture with similar objects in the Cookie-Theft picture is used for collecting the speech transcript, the use of any other pictures of different objects is likely to generate a different set of *n*-grams.

## Conclusion

We have proposed the combination of deep neural network and deep language models to predict MCI and AD-type dementia from sparse clinical language datasets. We learned deep language models using higher order *n*-gram vocabulary spaces. Experimental results show that the models predict MCI and AD-type dementia with less percentage error, perplexity, and statistically significant AUC on sparse clinical datasets. As part of our future work, we anticipate that our model has the potential to predict conversion from MCI to AD-type dementia [34]. We also see the potential for positive contribution to telemedicine by realizing an automated technique for the remote diagnosis or screening of MCI and AD-type dementia from a large population. We also plan to evaluate the D2NNLMs on large datasets and compare with other clinical measures.
