# Correction: Deep language space neural network for classifying mild cognitive impairment and Alzheimer-type dementia

**DOI:** 10.1371/journal.pone.0214103

**Published:** 2019-03-14

**Authors:** Sylvester Olubolu Orimaye, Jojo Sze-Meng Wong, Chee Piau Wong

There are errors in the Funding statement. The correct Funding statement is as follows: This study was funded in part by the Malaysian Ministry of Education via the Fundamental Research Grant Scheme (FRGS)-FRGS/2/2014/ICT07/MUSM/03/1. No additional external funding was received for this study.
